# Impact of a research methodology course emphasizing critical appraisal skills on dental students’ clinical decision-making self-efficacy: a single-arm educational intervention

**DOI:** 10.1186/s12909-026-08954-w

**Published:** 2026-04-13

**Authors:** Dhruv Ahuja, Areeba Parvez, Puneet Batra, Nidhin Philip Jose, Shravan Shetty, Gauri Kalra

**Affiliations:** 1 Department of Orthodontics and Dentofacial Orthopedics, Manav Rachna Dental College, Manav Rachna International Institute of Research and Studies(MRIIRS), Haryana, India; 2 Manav Rachna Dental College, Manav Rachna International Institute of Research and Studies(MRIIRS), Haryana, India; 3 Department of Orthodontics and Dentofacial Orthopaedics, Manipal College of Dental Sciences Mangalore, Manipal Academy of Higher Education, Manipal, India; 4 Department of Pedodontics and Preventive Dentistry, Manav Rachna Dental College, Manav Rachna International Institute of Research and Studies(MRIIRS), Haryana, India

**Keywords:** Dental education, Clinical decision-making, Research-oriented curriculum, Evidence-based practice

## Abstract

**Background:**

Undergraduate dental education in India emphasizes procedural competence, with limited structured exposure to research methodology and evidence appraisal. Research-oriented learning develops analytical reasoning, critical appraisal, and evidence interpretation skills that parallel core clinical reasoning processes and may strengthen students’ confidence in applying research evidence to patient care. The goals of this project were to implement a structured research methodology course emphasizing the critical appraisal process central to evidence-based practice (EBP), and assess its impact on clinical decision-making self-efficacy among undergraduate dental students.

**Methods:**

A prospective, single-center, single-arm quasi-experimental pre–post educational intervention study was conducted over 12 weeks among 250 undergraduate dental students. The research methodology course that comprised the education intervention was grounded in the principles of constructivism and competency-based education, incorporating blended learning, mentorship, and applied clinical scenarios. Clinical decision-making self-efficacy and research awareness were assessed using a validated 14-item questionnaire administered before and after the intervention. Statistical analyses included non-parametric pre–post comparisons, effect size estimation, subgroup analyses, and correlation testing. Ethical approval was obtained (MRDC/IEC/2025/58), and the study adhered to appropriate reporting guidelines for educational intervention research.

**Results:**

Clinical decision-making self-efficacy scores increased significantly from baseline to post-intervention (21.3 ± 3.8 to 28.9 ± 3.6; *p* < 0.001; Cohen’s *d* = 1.05). Research awareness scores also improved significantly (19.1 ± 4.2 to 27.4 ± 3.9; *p* < 0.001; Cohen’s *d* = 1.12). Improvements were observed across all academic years, with greater gains among senior students. Post-intervention feedback indicated high satisfaction and perceived clinical relevance.

**Conclusion:**

Implementation of a structured research methodology course emphasizing critical appraisal of evidence via active learning strategies was associated with improved clinical decision-making self-efficacy among dental students, supporting the integration of research training into undergraduate dental education.

**Supplementary Information:**

The online version contains supplementary material available at 10.1186/s12909-026-08954-w.

## Introduction

Dental education is undergoing a critical transformation as modern clinical practice increasingly demands evidence-based, patient-centered decision-making in addition to technical proficiency [1]. Traditional didactic models that emphasize procedural competence and passive, memorization-based learning are often insufficient to prepare undergraduate dental students for the complexity of contemporary healthcare environments [2].

Effective clinical decision-making now requires the ability to critically appraise scientific evidence, integrate research findings with clinical judgment, and adapt treatment strategies to individual patient contexts. Consequently, the integration of research-oriented training into undergraduate dental curricula has emerged as a strategic priority for advancing educational quality and clinical outcomes [3]. In India, undergraduate dental education is delivered through a structured curriculum that predominantly prioritizes clinical skill acquisition. However, formal and systematic exposure to research methodology, biostatistics, and evidence-based practice remains limited and inconsistently implemented across institutions [4].

Undergraduate dental students’ progress through staged clinical training, transitioning from preclinical simulation to supervised patient care in senior years. This developmental phase represents a critical period during which learners begin forming independent clinical reasoning patterns while still requiring structured academic support. Limited integration of research-oriented learning during this stage may restrict students’ ability to confidently interpret evidence and apply it within real-world clinical contexts [2, 3].

Although recent deliberations by regulatory bodies, have emphasized the need for curricular reform, outcome-driven integration of research-oriented learning at the undergraduate level remains inadequate [5]. This gap may constrain students’ ability to critically evaluate scientific literature, interpret emerging evidence, and confidently apply research findings to clinical decision-making. Evidence from health professions education suggests that research-oriented educational interventions can enhance analytical reasoning, critical thinking, and self-directed learning [6].

Early exposure to structured research training such as formulation of research questions, critical appraisal of literature, and interpretation of clinical data has been associated with improved engagement in evidence-based clinical practice [3]. Structured research training cultivates cognitive processes that directly parallel clinical reasoning. Framing research questions mirrors diagnostic problem formulation, critical appraisal of literature strengthens evaluation of evidence quality, and interpretation of research data reinforces analytical judgment under conditions of uncertainty. These higher-order skills—analysis, synthesis, and evaluative reasoning form the cognitive foundation of evidence-based clinical decision-making. Therefore, students’ acquisition of a critical appraisal framework may influence not only knowledge acquisition but also the reasoning processes underlying clinical judgment [7].

Importantly, clinical decision-making self-efficacy, derived from Bandura’s social cognitive theory, refers to learners’ belief in their capability to successfully perform tasks required for clinical reasoning and evidence application. According to this framework, self-efficacy develops through mastery experiences, observational learning, guided practice, and feedback. Educational strategies such as mentorship, case-based learning, and applied clinical scenarios components incorporated in the present curriculum are recognized mechanisms that strengthen self-efficacy by linking knowledge acquisition with experiential and social learning processes [8, 9].

Despite its educational relevance, empirical evidence examining the impact of structured research methodology coursework on clinical decision-making self-efficacy among undergraduate dental students, particularly within the Indian context, remains scarce [10]. Moreover, much of the existing literature in dental education focuses on knowledge acquisition, examination performance, or research output, with comparatively limited emphasis on students’ perceived competence and confidence in applying research evidence to real-world clinical scenarios. This represents a critical gap, as self-efficacy plays a central role in translating educational exposure into sustained behavioural change and meaningful clinical practice [11].

Rigorous evaluation of educational interventions that target both research competence and clinical decision-making confidence is therefore essential [12]. Against this backdrop, the present study was designed to develop, implement, and evaluate a structured research methodology course emphasizing critical appraisal processes for undergraduate dental students using a prospective pre–post quasi-experimental design. The primary objective was to assess changes in dental students’ clinical decision-making self-efficacy following exposure to a research methodology course focused on principles of evidence based practice and implemented via constructivist learning strategies. The hypothesis was that this research methods course would lead to a statistically significant improvement in clinical decision-making self-efficacy scores among undergraduate dental students. By linking pedagogical innovation to measurable educational outcomes, this study aims to generate evidence to inform curriculum development, guide institutional educational strategies, and support data-driven policy decisions for strengthening undergraduate dental education in India.

## Materials and methods

### Study design and setting

This study employed a prospective, single-center, single-arm Quasi experimental educational intervention design with a pre–post evaluation framework. The investigation was conducted at an accredited dental teaching institution- Manav Rachna Dental College, Delhi NCR, Faridabad, Haryana, India, over a 12-week academic period. The institution is recognized by the Dental Council of India and affiliated with Manav Rachna International Institute of Research and Studies, Delhi NCR, Faridabad, Haryana, India. The institution offers a structured undergraduate dental curriculum spanning five academic years, including preclinical foundational sciences followed by progressive clinical training under supervision. At the time of the study, approximately 300 undergraduate students were enrolled. The curriculum emphasizes didactic instruction and procedural clinical competence, while formal, structured training in research methodology and evidence-based clinical decision-making is limited in the early years of study.

The educational framework of the intervention was informed by constructivist learning theory, Bandura’s self-efficacy theory, and principles of evidence-based healthcare education. These frameworks emphasize active learner engagement, experiential learning, mentorship, and the integration of research evidence into clinical reasoning. The intervention structure was further aligned with competency domains recommended for undergraduate dental education, including critical appraisal skills, application of evidence to patient care, clinical reasoning, and reflective practice.

The competencies targeted in the curriculum were derived from:

1. established evidence-based dentistry competency models,
2. learning outcomes outlined in undergraduate dental education guidelines emphasizing research literacy and clinical decision-making, and
3. consensus from a multidisciplinary expert panel during curriculum development. These competencies focused on research question formulation, literature searching, appraisal of scientific evidence, interpretation of findings, and application of evidence to simulated clinical scenarios.

The study followed recommended reporting guidelines for non-randomized educational interventions (TREND statement)(Supplementary Table 1) [13]. As this study evaluated a new research course integrated into the institutional academic schedule, inclusion of a non-intervention control group was not feasible due to ethical and curricular uniformity requirements. Single-arm pre–post designs are commonly employed in educational implementation research to assess real-world curricular innovations. Nevertheless, the absence of a comparison group represents a limitation to internal validity, and findings should be interpreted as indicative of associations rather than causal effects. Ethical approval was obtained from the Institutional Ethics Committee(Ref No- MRDC/IEC/2025/58), and written informed consent was secured from all participants prior to enrolment.

Participation was entirely voluntary and had no influence on academic evaluation, grading, or faculty appraisal. Faculty mentors involved in teaching were not involved in data analysis to minimize perceived coercion. Questionnaires were anonymized using unique codes, and no identifying information was stored with responses. Data were securely stored on password-protected institutional systems accessible only to the research team. The 12-week duration was selected to align with one academic teaching cycle and to allow progressive skill development, mentorship interaction, and reinforcement of research and evidence-based learning concepts. Sustained, multi-session educational exposure is considered more effective than short-term workshops for influencing higher-order outcomes such as self-efficacy and application of evidence, while remaining feasible within the routine academic schedule [5, 14].

### Participants

**Fig. 1:**
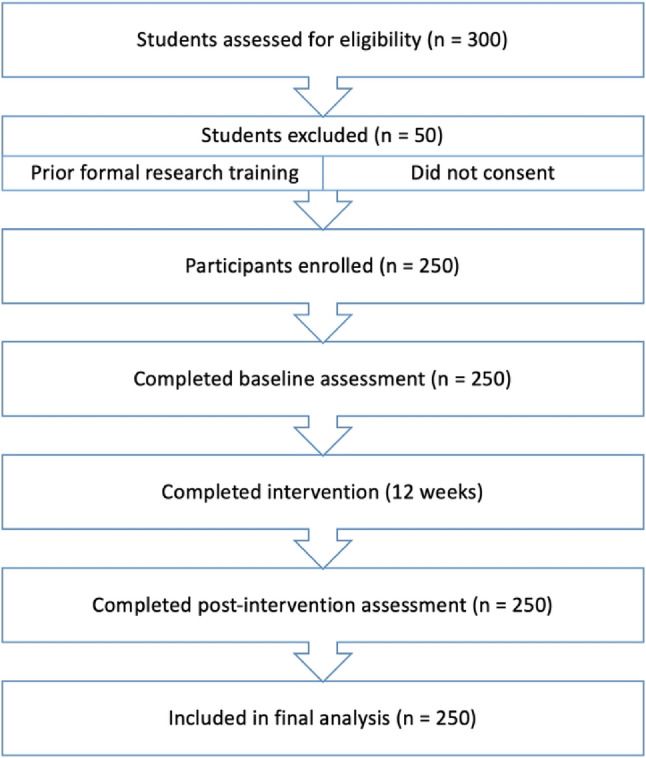
Participant flow diagram showing screening, exclusions, enrollment, completion of the 12-week intervention, and inclusion in final analysis (*n* = 250)

Participants were undergraduate dental students from the first to third academic years recruited through institutional academic notification. In the local curriculum context, formal structured training in research methodology and biostatistics is not systematically delivered in early undergraduate years; exposure, when present, is typically limited to brief theoretical lectures without applied components. Therefore, prior formal research training was uncommon but assessed to reduce potential baseline heterogeneity. Students were eligible if they were actively enrolled and provided informed consent. Students with documented participation in structured research training programs, certificate courses in research methodology/biostatistics, or externally funded research internships were excluded to reduce confounding due to advanced prior exposure. Informal exposure (e.g., attendance at seminars, occasional faculty discussions, or non-structured activities) was not considered an exclusion criterion, as such exposure is common and unlikely to produce substantial competency differences. Institutional academic approval was obtained to conduct the intervention as a curriculum-enrichment activity integrated within scheduled academic hours. Participation was voluntary and did not influence academic evaluation. Baseline academic year and cumulative GPA were recorded and incorporated into subgroup and stratified analyses to account for variability in prior academic experience. While residual differences in informal exposure cannot be entirely excluded, this reflects real-world educational diversity and enhances ecological validity rather than introducing systematic allocation bias. Participants represented early to intermediate stages of clinical exposure, transitioning from preclinical training to supervised patient care. To address heterogeneity in baseline clinical experience, academic year was recorded and incorporated into subgroup and stratified analyses (Fig. 1).

### Sample size

Sample size estimation was performed using G*Power software (version 3.1.7) based on a repeated-measures design. Assuming a medium effect size derived from prior educational intervention studies in health professions education, a two-tailed alpha level of 0.05, and 80% statistical power, the minimum required sample size was 210 participants. To account for an anticipated attrition rate of approximately 15–20%, a target sample of 250 undergraduate dental students was planned and achieved. Participants were drawn from three academic strata: first-year, second-year, and third-year undergraduate dental students. All eligible students within these academic years were invited through institutional academic notification, and recruitment followed a census-based approach rather than random sampling. Students who consented and met eligibility criteria were enrolled until the target sample size was reached. This approach ensured representation across stages of clinical exposure while reflecting real-world educational implementation.

### Curricular context

Undergraduate dental education follows a progression from preclinical, didactic learning in early years to supervised clinical training in senior years. Although clinical exposure increases over time, structured training in research methodology, critical appraisal, and application of evidence to clinical decision-making remains limited and largely theoretical within the routine curriculum. This gap provided the educational rationale for introducing a structured research-oriented learning module.

### Educational intervention framework

The intervention was delivered as a structured educational module integrated alongside the regular academic schedule over a 12-week period, without replacing existing coursework. Students from all participating academic years received the same core curriculum content, instructional sessions, mentorship exposure, and practical activities. The educational framework was developed based on constructivist learning theory and principles of evidence-based education, emphasizing active learner engagement, mentorship, and clinical application of research evidence. A multidisciplinary faculty team collaboratively designed the curriculum to ensure pedagogical relevance and clinical applicability [5, 14]. The curriculum followed a structured educational pathway: Inputs (faculty mentorship, instructional modules, digital resources); Learning Activities (flipped classrooms, workshops, database exercises, simulated clinical scenarios); Immediate Outputs (research literacy and evidence appraisal skills); Educational Outcomes (clinical decision-making self-efficacy and research application). The 12-week duration was selected to align with the institutional academic term structure and to allow staged exposure to course components without overloading students. Sessions were scheduled during designated academic enrichment hours and did not replace or reduce core curricular teaching time. The intervention was reviewed and permitted at the institutional academic level as a supplementary educational activity, ensuring compatibility with routine semester requirements [15, 16].

### Baseline assessment

At baseline, participants completed a content-validated and pilot-tested clinical decision-making self-efficacy questionnaire, which assessed multiple domains including evidence appraisal, diagnostic reasoning, confidence in treatment planning, and research orientation in clinical decision-making. The questionnaire was administered in a supervised setting to ensure uniformity of data collection and minimize response bias. The questionnaire comprised 14 items rated on a 5-point Likert scale (1 = strongly disagree to 5 = strongly agree), yielding a total possible score range of 14–70. For analytical clarity, domain-specific scores were calculated and analyzed separately for clinical decision-making self-efficacy (7 items; score range 7–35) and research awareness and application (7 items; score range 7–35) [9–11] (Supplementary Table 2). The tool was designed to align with the dual focus of the intervention: clinical decision-making self-efficacy and research awareness/application.

### Curriculum development and content

The curriculum consisted of structured instructional modules designed to enhance both clinical decision-making and research-oriented skills. Modules covered fundamental research methodology, framing clinical questions, literature searching, evaluating scientific evidence, and basic biostatistics. Instruction was delivered through a combination of flipped classrooms, hybrid online and in-person lectures, hands-on database exercises using PubMed, and workshops on managing references and performing basic statistical analyses in SPSS and Excel. Curriculum content and sequencing were reviewed by a multidisciplinary expert panel specialists from orthodontics, community dentistry, and health research to ensure pedagogical validity and applicability across dental disciplines. The curriculum was not discipline-specific but focused on generalizable competencies in evidence appraisal, research literacy, and clinical decision-making relevant to all areas of undergraduate dental practice.

Students were organized into small mentorship groups of five to six participants, each supervised by a trained faculty mentor. Weekly mentorship sessions provided formative feedback, encouraged reflective learning, and monitored academic progress throughout the 12-week intervention. Practical exercises and simulated clinical scenarios were incorporated to facilitate the application of evidence to clinical decision-making [17, 18].

### Implementation phase

The intervention was implemented over a 12-week period in a sequential manner. The initial phase focused on orientation and foundational research principles, followed by structured training in literature searching, critical appraisal, and research question development. Subsequent sessions emphasized research design concepts and applied biostatistics. In the final phase, participants engaged in simulated clinical scenarios requiring integration of research evidence into clinical decision-making. Continuous formative assessments, including short quizzes, attendance monitoring, structured group activity rubrics, and standardized faculty feedback, were employed to reinforce learning outcomes.

### Post-Intervention assessment

Upon completion of the curriculum, participants were re-assessed using the same Clinical Decision-Making Self-Efficacy Questionnaire to evaluate changes attributable to the intervention. Additional post-intervention Likert-scale items (5 items) assessed participants’ perceptions of curriculum relevance, clinical applicability, and satisfaction. Adverse educational, psychological, or academic effects were monitored via post-intervention questions asking participants to report perceived stress, academic burden, or any negative impact on coursework; no formal scoring was conducted. Open-ended feedback was also collected to capture qualitative insights regarding learning experience and challenges.

### Instrument validation and reliability

The questionnaire was adapted from previously validated instruments assessing clinical decision-making self-efficacy and research orientation. The instrument comprised three conceptual sections: (1) Clinical Decision-Making Self-Efficacy (Items 1–7), adapted from existing self-efficacy frameworks in clinical education; (2) Research Awareness and Application (Items 8–14), developed to assess research literacy and evidence appraisal skills relevant to clinical reasoning; and (3) Attitudes and Feedback (post-only Items 15–19), designed to evaluate perceived educational relevance and learner satisfaction. Items in Sects. 2 and 3 were developed de novo to address curriculum-specific learning objectives and were included following expert panel validation [19].

Content validity was confirmed via expert review by a multidisciplinary panel (orthodontist, community dentist, community medicine specialist, psychologist, research director), with a scale-level Content Validity Index (S-CVI/Ave) of 0.89, indicating excellent content validity. A pilot study with 30 undergraduate dental students assessed clarity, feasibility, and comprehensibility, and minor refinements were made based on feedback. Open-ended items captured descriptive learner perspectives and were summarized narratively rather than formally analyzed [20, 21].

Internal consistency reliability was assessed for the pre- and post-intervention assessments combined using Cronbach’s alpha, with coefficients ≥ 0.70 across all domains, indicating acceptable to excellent reliability. Separate calculations for pre- and post-assessments were not performed, as the combined analysis reflects overall instrument reliability across the study period. Formal construct validity testing (e.g., exploratory or confirmatory factor analysis) and responsiveness assessment were not conducted, representing a measurement limitation.

### Statistical analysis

Statistical analysis was performed using IBM SPSS Statistics (version 29, IBM Corp., Armonk, NY, USA**)**. Descriptive statistics were calculated and presented as means with standard deviations, medians with interquartile ranges, or frequencies and percentages, as appropriate. Data normality was assessed using the Shapiro–Wilk test. Although non-parametric tests were applied due to minor deviations from normality, the large sample size permits reporting of means and standard deviations, which are considered robust descriptive estimates under the Central Limit Theorem. Given deviations from normality in several outcome variables, pre–post comparisons were conducted using non-parametric tests as appropriate. Comparisons across academic years were performed using one-way analysis of variance or Kruskal–Wallis tests, as appropriate. To explore the influence of potential confounders such as academic year and cumulative GPA, stratified subgroup analyses were performed. Effect sizes were calculated using Cohen’s d, defined as the mean pre–post difference divided by the pooled standard deviation of pre- and post-intervention scores. Cohen’s d values of 0.2, 0.5, and 0.8 were interpreted as small, medium, and large effects, respectively. Pearson correlation coefficients (r) were classified as weak (0.10–0.29), moderate (0.30–0.49), and strong (≥ 0.50). Cohen’s d was reported to facilitate interpretation of practical significance despite non-normal distributions. Partial eta squared (η²ₚ) was reported for repeated-measures comparisons where applicable. Associations between academic performance and post-intervention outcomes were examined using Pearson’s correlation coefficients. Statistical significance was set at *p* < 0.05. Missing data were minimal (< 5%) and assessed for patterns. As missingness appeared random and limited in magnitude, complete-case paired analyses were conducted without imputation, consistent with recommended practice for low-level missing data. Paired analyses included only participants with complete pre–post data.

## Results

### Sample characteristics

A total of 250 undergraduate dental students participated in the study across three academic years. The mean age of participants was 21.4 ± 1.2 years, with females comprising 64% (*n* = 160) and males 36% (*n* = 90). Academic year distribution included 80 (32%) first-year, 100 (40%) second-year, and 70 (28%) third-year students (Table 1). For the purposes of this study, prior formal, structured training was defined as completion of a systematically organized course or program providing comprehensive instruction in research methodology, including framing clinical questions, literature searching, critical appraisal of scientific evidence, and basic biostatistics, which is more extensive than the informal exposure typically gained through ad hoc workshops, project assistance, or brief research activities. Students with such formal prior training were excluded to ensure baseline comparability.

**Table 1:** Baseline Demographic and Academic Characteristics of Participants (*n* = 250)

Variable	Category	Frequency (*n*)	Percentage (%)
Gender	Male	90	36.0
	Female	160	64.0
Academic Year	First Year	80	32.0
	Second Year	100	40.0
	Third Year	70	28.0
Prior Research Exposure	Yes	78	31.2
	No	172	68.8
Previous Research Project Participation	Yes	92	36.8
	No	158	63.2
Currently Involved in Research Activities	Yes	55	22.0
	No	195	78.0

However, a majority of participants reported informal or ad hoc exposure to research activities: 78 students (31.2%) reported some prior research exposure, 92 students (36.8%) had participated in a research project, and 55 students (22%) were actively involved in research-related activities at the time of enrolment. Prior informal research exposure was measured at baseline and included as a subgroup variable in stratified analyses to account for potential differences in research familiarity. All eligible students from the first to third academic years were invited to participate, and the distribution of participants across academic years closely reflected the institutional enrollment pattern, supporting representativeness of the study sample. Missing pre–post data were minimal (< 5%) and showed no systematic pattern; complete-case paired analyses were conducted without imputation.

### Pre– and post–intervention outcomes

A statistically significant improvement in clinical decision-making self-efficacy subdomain scores was observed following completion of the research-oriented curriculum, with mean scores increasing from 21.3 ± 3.8 at baseline to 28.9 ± 3.6 post-intervention (*p* < 0.001; Cohen’s d = 1.05).

**Fig. 2:**
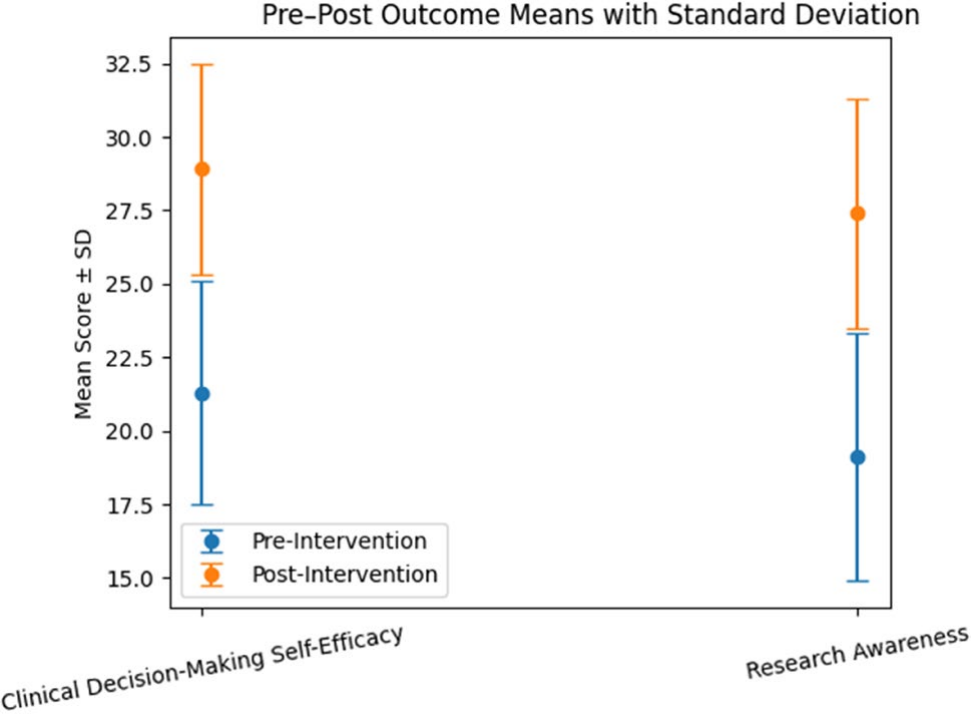
Comparison of mean scores (± SD) for Clinical Decision-Making Self-Efficacy and Research Awareness before and after intervention

Similarly, research awareness and application subdomain scores demonstrated a significant increase from 19.1 ± 4.2 pre-intervention to 27.4 ± 3.9 post-intervention (*p* < 0.001; Cohen’s d = 1.12). These findings indicate a substantial positive association between participation in the curriculum and improvements in clinical decision-making confidence and research orientation. Pre- and post-intervention outcomes are presented in Table 2; Fig. 2.

**Table 2:** Pre– and Post–Intervention Comparison of Primary Outcomes (*n* = 250)

Outcome Domain	Pre-Intervention Mean ± SD	Post-Intervention Mean ± SD	Statistical Test	*p*-value	Effect Size
Clinical Decision-Making Self-Efficacy	21.3 ± 3.8	28.9 ± 3.6	Wilcoxon signed-rank	< 0.001	Cohen’s d = 1.05
Research Awareness	19.1 ± 4.2	27.4 ± 3.9	Wilcoxon signed-rank	< 0.001	Cohen’s d = 1.12

### Subgroup analyses

Subgroup analyses were pre-specified to explore potential variation related to academic year (as a proxy for clinical exposure), prior research exposure, gender, and GPA, which may influence baseline self-efficacy levels. Subgroup analyses using Mann–Whitney U and Kruskal–Wallis tests revealed a statistically significant difference in score gains across academic years (*p* = 0.021), with third-year students demonstrating a greater magnitude of improvement in clinical decision-making self-efficacy compared to first- and second-year students.

Students with prior informal research exposure exhibited higher baseline scores compared to those without such exposure (mean 23.1 vs. 20.4, *p* < 0.05). However, both groups demonstrated statistically significant improvements following the intervention, and no statistically significant difference was observed in the magnitude of post-intervention score gains between the groups. No statistically significant difference in improvement was observed between male and female participants (*p* = 0.18). Subgroup comparisons are summarized in Table 3. Adjustment for multiple comparisons was not performed; therefore, subgroup findings should be interpreted as exploratory.

**Table 3:** Subgroup Analyses of Clinical Decision-Making Score Changes

Variable	Comparison Groups	*p*-value
Academic Year	1st vs. 2nd vs. 3rd Year	0.021
Prior Research Exposure	Yes vs. No	0.043
Gender	Male vs. Female	0.18

Between-group comparisons of pre–post change scores were performed using non-parametric tests. H denotes the Kruskal–Wallis test statistic, and U denotes the Mann–Whitney U statistic

### Correlation analysis

Correlation analyses were conducted to explore associations between academic background variables and post-intervention outcomes. Cumulative GPA demonstrated a moderate positive correlation with post-intervention clinical decision-making self-efficacy scores (*r* = 0.42, *p* < 0.01), indicating that students with stronger academic performance tended to report higher confidence following the intervention. Prior research exposure showed a weak-to-moderate positive association with post-intervention scores (point-biserial rₚ_β_ = 0.31, *p* = 0.02), suggesting that previous familiarity with research activities may have modestly enhanced students’ ability to benefit from the curriculum. However, the magnitude of these correlations indicates that academic factors explained only a limited proportion of outcome variability, implying that the observed improvements were not solely dependent on prior academic performance or research experience. These findings support the applicability of the curriculum across diverse learner profiles (Table 4).

**Table 4:** Correlation Between Academic Variables and Post-Intervention Outcomes

Variable	Correlated Outcome	Correlation Coefficient	Strength	*p*-value
Cumulative GPA	Post-intervention clinical decision-making score	*r* = 0.42	Moderate positive	< 0.01
Prior Research Exposure	Post-intervention clinical decision-making score	rₚ_β_ = 0.31	Weak–moderate positive	0.02

*r*  Pearson correlation coefficient, *rₚ*_β_ point-biserial correlation coefficient

### Post-intervention perceptions and feedback

Post-intervention feedback indicated high levels of participant satisfaction with the curriculum. A total of 92% of students agreed or strongly agreed that the program improved their understanding of evidence-based dentistry, while 89.2%reported increased confidence in applying clinical decision-making skills. Hands-on research activities were perceived as beneficial by 84.8% of participants.

**Fig. 3:**
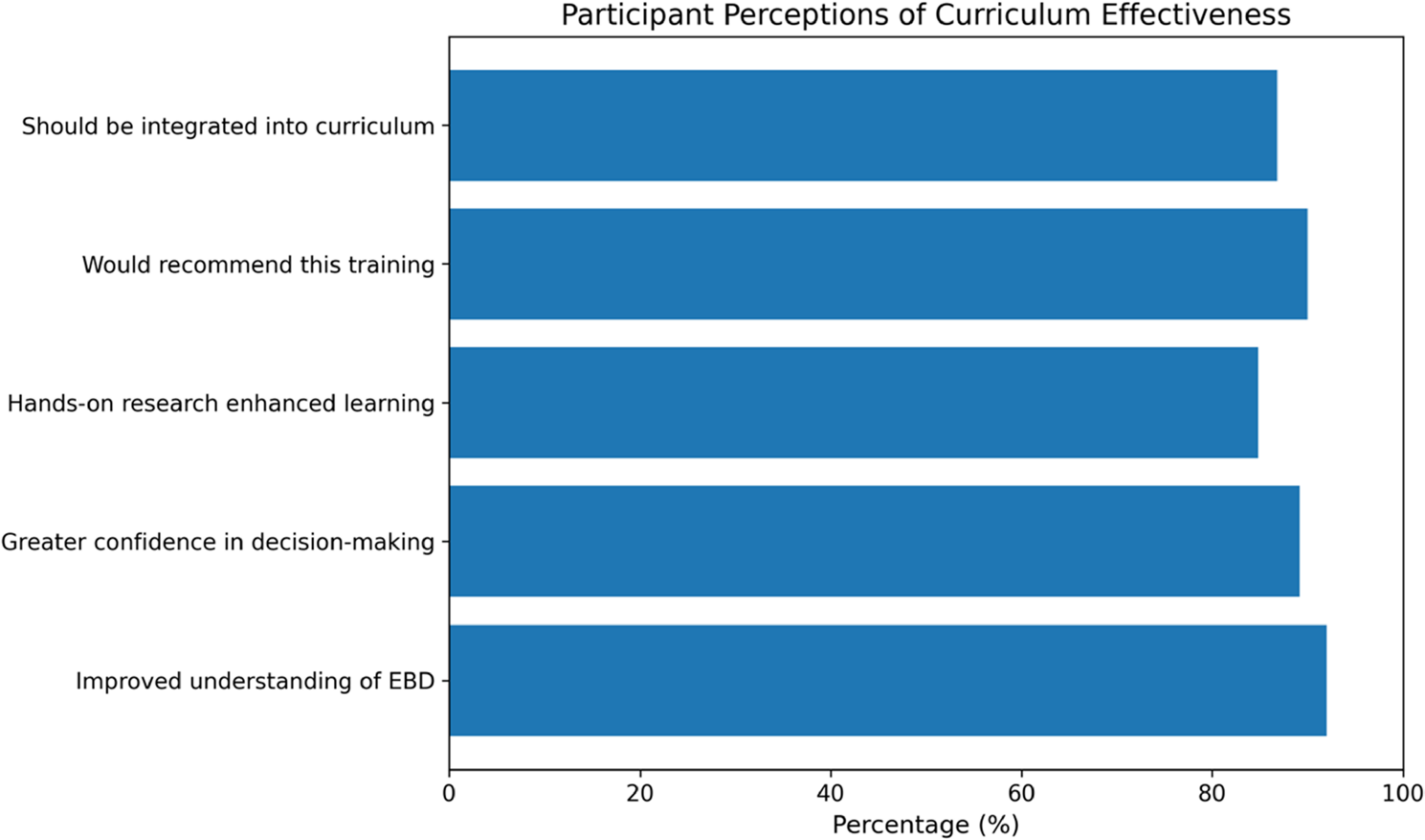
Post-Intervention Perceptions of the Curriculum

Despite these positive perceptions, commonly reported challenges included time constraints, balancing academic workload, and initial unfamiliarity with statistical software. Importantly, 86.8% of participants recommended integrating the research-oriented curriculum into the regular undergraduate dental program. Participant feedback is summarized in Table 5; Fig. 3. Open-ended responses were reviewed to contextualize quantitative findings, with common comments reflecting improved confidence in evidence-based decision-making and challenges related to time and statistics.

**Table 5:** Post-Intervention Participant Perceptions of the Curriculum (*n* = 250)

Statement	Agree / Strongly Agree (*n*)	Percentage (%)
The curriculum improved my understanding of evidence-based dentistry	230	92.0
I feel more confident in applying clinical decision-making after the program	223	89.2
Hands-on research experience enhanced my learning	212	84.8
I would recommend this research training to others	225	90.0
This module should be integrated into the regular BDS curriculum	217	86.8

Responses reflect “Agree” and “Strongly Agree” combined

## Discussion

The integration of structured research training into undergraduate dental education is increasingly recognized as important for supporting the development of critical thinking, clinical reasoning, and engagement with evidence-based decision-making processes. In the present study, implementation of a systematically designed research-oriented curriculum resulted in a statistically significant and educationally meaningful improvement in clinical decision-making self-efficacy among undergraduate dental students. While the large observed effect size suggests a substantial improvement in students’ perceived abilities, interpretation should be cautious given the single-group pre–post design and absence of a comparison group.

The relatively low baseline clinical decision-making self-efficacy scores observed in this cohort (mean 21.3 on a 12–60 scale) warrant specific consideration. This finding is plausible within the Indian undergraduate dental education context, where early curricular emphasis is predominantly placed on procedural competence and examination-oriented learning, with limited formal exposure to research methodology, critical appraisal, or evidence-based practice [4]. As a result, students may possess foundational clinical knowledge yet lack confidence in independently interpreting scientific literature or applying research evidence to clinical decisions. Similar low baseline self-efficacy levels have been reported in studies evaluating evidence-based practice readiness among undergraduate health professions students prior to structured research training, supporting the validity of this observation rather than suggesting a measurement artifact [22].

Following the intervention, the marked increase in self-efficacy scores reflects meaningful educational gain and suggests that targeted research-oriented curricula may support students’ confidence and readiness to engage in evidence-informed clinical reasoning. These findings align with previous reports demonstrating that early exposure to research skills enhances analytical reasoning, promotes self-directed learning, and strengthens confidence in evidence-based clinical decision-making. Importantly, by focusing on self-efficacy rather than knowledge acquisition alone, the present study captures an outcome associated with confidence and readiness for evidence-based practice, although not a direct measure of clinical competence [23]. However, improvements in self-efficacy should not be interpreted as direct evidence of enhanced clinical competence, as performance-based outcomes were not assessed. Subgroup analyses revealed that third-year students exhibited greater improvements compared to their junior counterparts. This trend likely reflects the synergistic effect of concurrent clinical exposure and research training, wherein students with greater clinical experience are better positioned to contextualize research evidence within real-world patient scenarios. Students with prior informal research exposure demonstrated higher baseline self-efficacy; however, both groups experienced comparable gains following the intervention, suggesting that the curriculum was effective across varying levels of prior experience. These findings highlight the adaptability of the curriculum and support its potential scalability across different stages of undergraduate training [24].

Qualitative feedback further reinforced the quantitative outcomes, with a substantial majority of participants reporting improved confidence and perceived relevance of evidence-based dentistry. Hands-on components such as database searching, reference management, and introductory statistical analysis were consistently identified as particularly valuable. This emphasizes the importance of experiential learning and mentorship in translating abstract research concepts into learning experiences relevant to clinical reasoning processes. The mentorship-based, small-group format likely contributed to sustained engagement and individualized feedback, enhancing learning outcomes [25].

The strengths of this study include its theory-informed curriculum design, phased implementation, blended instructional strategies, validated outcome measures, and robust statistical analysis. Nevertheless, certain limitations must be acknowledged. Additionally, outcomes were assessed immediately post-intervention, precluding conclusions regarding long-term retention of self-efficacy gains or translation into observable clinical behavior. Although self-efficacy is a recognized predictor of practice change, future studies should incorporate objective clinical performance measures and longitudinal follow-up.

### Clinical and educational significance

This study provides empirical evidence that structured research-oriented curricula can enhance perceived clinical decision-making self-efficacy in undergraduate dental students, potentially supporting readiness for evidence-based practice. By fostering confidence, analytical reasoning, and research literacy, such interventions may help prepare students to engage more confidently with evidence-informed clinical reasoning, while objective competence must be established through performance-based assessment and support curricular reforms aimed at producing graduates capable of integrating research evidence into clinical care. The findings emphasize the value of mentorship, hands-on research activities, and early exposure to research methodology as key components of effective dental education [26].

### Limitations

This study include the single-center design, absence of a control group, and reliance on self-reported self-efficacy measures. Additionally, well-recognized threats to internal validity in single-group pre–post designs such as maturation, testing effects from repeated questionnaire exposure, and response-shift bias in self-perception may have contributed to observed score changes independent of the intervention. As the instrument was partially adapted and partially newly developed, findings should be interpreted as measuring perceived competence rather than objective clinical performance, and future studies incorporating multi-institutional designs, longitudinal follow-up, and objective clinical performance assessments are warranted to evaluate the durability and real-world applicability of self-efficacy gains. Additionally, the short-term nature of the assessment precludes conclusions regarding long-term behavior change or actual patient care outcomes.

### Future scope and educational implications

This study advances dental education research at theoretical, methodological, and contextual levels. It applies self-efficacy theory to research training outcomes, evaluates a structured 12-week curriculum model within undergraduate dental education, and provides empirical evidence from an Indian educational context where such evaluations are limited. Together, these findings contribute to understanding how curriculum-level research training may influence readiness for evidence-based clinical decision-making.

Future research should focus on multi-institutional randomized or controlled designs to validate these findings across diverse educational settings. Longitudinal studies evaluating sustained self-efficacy, clinical decision-making performance, and scholarly engagement would provide valuable insight into the durability and real-world impact of research-oriented curricula. Integration of such curricula into formal undergraduate programs, rather than as supplementary interventions, may further strengthen evidence-based practice readiness among graduating dentists. At a policy level, these findings support the inclusion of structured research training as a core component of undergraduate dental education in India.

## Conclusion

The present study demonstrates that a structured, research-oriented curriculum can significantly enhance clinical decision-making self-efficacy among undergraduate dental students. By addressing a critical gap in traditional dental education, such curricula have the potential to support the development of learners who report greater confidence and readiness to engage with evidence in clinical reasoning contexts. These findings provide empirical support for curriculum reform and underscore the value of integrating research-oriented training into undergraduate dental education to strengthen both educational and clinical outcomes.

## Supplementary Information

Supplementary Material 1.

Supplementary Material 2.

## Data Availability

The datasets used and/or analyzed during the current study available from the corresponding author on reasonable request.

## References

[1] Lee JI. Dental education now and in the future. J Periodontal Implant Sci. 2023;53(3):171–2. 10.5051/jpis.235303edi01. 37387125 10.5051/jpis.235303edi01 PMC10315257

[2] Aulakh J, Wahab H, Richards C, Bidaisee S, Ramdass PVAK. Self-directed learning versus traditional didactic learning in undergraduate medical education: a systemic review and meta-analysis. BMC Med Educ. 2025;25(1):70. 10.1186/s12909-024-06449-0. 39815233 10.1186/s12909-024-06449-0 PMC11737201

[3] Kumah EA, McSherry R, Bettany-Saltikov J, van Schaik P, Hamilton S, Hogg J, Whittaker V. Evidence-informed practice versus evidence-based practice educational interventions for improving knowledge, attitudes, understanding, and behavior toward the application of evidence into practice: A comprehensive systematic review of UG student. Campbell Syst Rev. 2022;18(2):e1233. 10.1002/cl2.1233. 36911346 10.1002/cl2.1233 PMC9013402

[4] Priyank H, Mohan S, Viswanath B, Kumar G, Prasad R. Knowledge of undergraduate Indian dental students on research methodology. Bioinformation. 2025;21(1):48–53. 10.6026/973206300210048. 40255308 10.6026/973206300210048 PMC12008794

[5] Deshpande AN, Mathur VP, Lele GS, Nirmal L, Saha S, Muthu MS, Marwah N, Khanna R, Anandakrishna L. Identifying Needs and Preparing for Curriculum Changes in Indian Dental Education. Int J Clin Pediatr Dent. 2024;17(7):842–50. 10.5005/jp-journals-10005-2846. 39372520 10.5005/jp-journals-10005-2846 PMC11451925

[6] Wilkes S, Maggio LA, Martin PC, Melton J, Zheng B. Self-Directed Learning in Health Professions Education: A Systematic Review and Meta-Analysis. Perspect Med Educ. 2026;15(1):37–52. 10.5334/pme.2128. 41626409 10.5334/pme.2128 PMC12857615

[7] Leal P, Poeira A, Mendes DA, Batalha N, Franco H, Nunes L, Marques F, Pađen L, Stefaniak M, Pérez-Perdomo A, Bangels L, Lemmens K, Amaral G. Teaching and Learning Clinical Reasoning in Nursing Education: A Student Training Course. Healthc (Basel). 2024;12(12):1219. 10.3390/healthcare12121219. 10.3390/healthcare12121219 PMC11202887 38921333

[8] Bandura A. Reflections on self-efficacy. Adv Behav Res Therapy. 1978;1:237–69. 10.1016/0146-6402(78)90012-7.

[9] Bandura A. The explanatory and predictive scope of self-efficacy theory. J Soc Clin Psychol. 1986a;4:359–73. 10.1521/jscp.1986.4.3.359.

[10] Aboalrob W, Ayed A, Malak MZ, Aqtam I. Understanding the influence of self-concept on clinical decision-making among nurses: A cross-sectional study. PLoS ONE. 2025;20(8):e0330905. 10.1371/journal.pone.0330905. 40853911 10.1371/journal.pone.0330905 PMC12377617

[11] Ankowski J, Krokosz S, Zięba S, et al. Self-assessed preparedness of final-year dental students and dental interns in Poland: a multi-institutional study. BMC Med Educ. 2025;25:627. 10.1186/s12909-025-07215-6. 40296009 10.1186/s12909-025-07215-6 PMC12038962

[12] Elmanaseer WR, Al-Omoush SA, Alamoush RA, Abu Zaghlan R, Alsoleihat F. Dental Students’ Perception and Self-Perceived Confidence Level in Key Dental Procedures for General Practice and the Impact of Competency Implementation on Their Confidence Level, Part I (Prosthodontics and Conservative Dentistry). Int J Dent. 2023;2023:2015331. 10.1155/2023/2015331. 37868108 10.1155/2023/2015331 PMC10586436

[13] Des Jarlais DC, Lyles C, Crepaz N, TREND Group. Improving the reporting quality of nonrandomized evaluations of behavioral and public health interventions: the TREND statement. Am J Public Health. 2004;94(3):361–6. 10.2105/ajph.94.3.361. 14998794 10.2105/ajph.94.3.361 PMC1448256

[14] atelarou AE, Mechili EA, Ruzafa-Martinez M, Dolezel J, Gotlib J, Skela-Savič B, Ramos-Morcillo AJ, Finotto S, Jarosova D, Smodiš M, Mecugni D, Panczyk M, Patelarou E. Educational Interventions for Teaching Evidence-Based Practice to Undergraduate Nursing Students: A Scoping Review. Int J Environ Res Public Health. 2020;17(17):6351. 10.3390/ijerph17176351. 32878256 10.3390/ijerph17176351 PMC7503534

[15] Wilesmith S, Mandrusiak A, Lang R, Martin R, Lu A, Forbes R. Educational Interventions to Develop and Enhance Clinical Documentation Skills in Health Professional Students: A Systematic Review. Clin Teach. 2025;22(5):e70157. 10.1111/tct.70157. 40755057 10.1111/tct.70157 PMC12319399

[16] Yüksel EM, Green CS, Vlach HA. Durability of Students’ Learning Strategies Use and Beliefs Following a Classroom Intervention. Behav Sci (Basel). 2025;15(5):706. 10.3390/bs15050706. 40426483 10.3390/bs15050706 PMC12108934

[17] Wadgave U, Khairnar MR, Kadu TS, Chadha GK, Wadgave Y. Effect of training on evidence-based practice to undergraduate dental students: pre and postexperimental study. Int J Evid Based Healthc. 2020;18(1):101–7. 10.1097/XEB.0000000000000199. 31335664 10.1097/XEB.0000000000000199

[18] Goyal T, Ahuja D, Rana A, Mallick S, Bairwa U. Influence of dental aesthetics on psychosocial well-being in the North Indian population. Pesqui Bras Odontopediatria Clin Integr. 2025;26:e250020.

[19] Axboe MK, Christensen KS, Kofoed PE, Ammentorp J. Development and validation of a self-efficacy questionnaire (SE-12) measuring the clinical communication skills of health care professionals. BMC Med Educ. 2016;16(1):272. 10.1186/s12909-016-0798-7. 27756291 10.1186/s12909-016-0798-7 PMC5069791

[20] Shi J, Mo X, Sun Z. Zhong Nan Da Xue Xue Bao Yi Xue Ban. 2012;37(2):152–5. 10.3969/j.issn.1672-7347.2012.02.007. 22561427 10.3969/j.issn.1672-7347.2012.02.007

[21] Dalawi I, Isa MR, Chen XW, Azhar ZI, Aimran N. Development of the Malay Language of understanding, attitude, practice and health literacy questionnaire on COVID-19 (MUAPHQ C-19): content validity & face validity analysis. BMC Public Health. 2023;23(1):1131. 10.1186/s12889-023-16044-5. 37312175 10.1186/s12889-023-16044-5 PMC10262113

[22] Alhejaili AA, Alshahrani B, Muslihi A, Garcia PRB, Roque MY, Alharbi RS, Fadlalmola HA. Nursing Students’ Satisfaction and Self-Confidence After Short-Term Clinical Preparation: A Cross-Sectional Study. Nurs Rep. 2025;15(9):317. 10.3390/nursrep15090317. 41003272 10.3390/nursrep15090317 PMC12472800

[23] Reddy R, Ainsworth OM, Gray MJ, Hildebrand A, Waugh J, Lindwall J, Huerta J, Keller TE. Evaluating gains in student self-efficacy in scientific literacy associated with a brief curricular intervention. J Microbiol Biol Educ. 2025;26(3):e0012625. 10.1128/jmbe.00126-25. 40932270 10.1128/jmbe.00126-25 PMC12687605

[24] Regmi A, Mao X, Qi Q, Tang W, Yang K. Students’ perception and self-efficacy in blended learning of medical nutrition course: a mixed-method research. BMC Med Educ. 2024;24(1):1411. 10.1186/s12909-02406339-5. 39627743 10.1186/s12909-024-06339-5 PMC11616338

[25] Chai HH, Gao SS, Chen KJ, Duangthip D, Lo ECM, Chu CH. A Concise Review on Qualitative Research in Dentistry. Int J Environ Res Public Health. 2021;18(3):942. 10.3390/ijerph18030942. 33499023 10.3390/ijerph18030942 PMC7908600

[26] Al Sweleh FS. Integrating scientific research intoundergraduate curriculum: A new direction in dental education. J Health Spec. 2016;4:42–5. 10.4103/1658-600X.173845.

