# Supplementary material for: Impact of a research methodology course emphasizing critical appraisal skills on dental students’ clinical decision-making self-efficacy: a single-arm educational intervention
Source: BMC Med Educ. 2026 Apr 13;26:849. doi: 10.1186/s12909-026-08954-w (PMC13217952; doi:10.1186/s12909-026-08954-w)
Supplement: Supplementary file 1 — Supplementary Material 1. [file 12909_2026_8954_MOESM1_ESM.docx]

**Clinical Decision-Making and Research Orientation Questionnaire**

**(Pre- and Post-Intervention Tool)**

**Section A: Demographic Details**

1. Name (optional): ____________________
2. Age: ______
3. Gender: ☐ Male ☐ Female ☐ Other
4. Year of BDS Study: ☐ 3rd Year ☐ Final Year ☐ Intern
5. Have you received any formal training in research methodology before? ☐ Yes ☐ No
6. Have you participated in any research project previously? ☐ Yes ☐ No
7. Are you currently involved in any academic writing (case reports, reviews, etc.)? ☐ Yes ☐ No

**Section B: Clinical Decision-Making Self-Efficacy (Likert Scale: 1–Strongly Disagree to 5–Strongly Agree)**

|  | **Statement** | **Rating (1–5)** |
| --- | --- | --- |
| 1 | I feel confident making clinical decisions based on patient symptoms and findings. | ☐☐☐☐☐ |
| 2 | I can interpret clinical and diagnostic data effectively. | ☐☐☐☐☐ |
| 3 | I can link patient complaints to evidence-based solutions. | ☐☐☐☐☐ |
| 4 | I am confident in selecting appropriate treatment options for hypothetical cases. | ☐☐☐☐☐ |
| 5 | I understand how to differentiate between high- and low-quality clinical evidence. | ☐☐☐☐☐ |
| 6 | I feel prepared to engage in clinical discussions involving literature support. | ☐☐☐☐☐ |
| 7 | I can use PubMed or other databases to find relevant clinical studies. | ☐☐☐☐☐ |

**Section C: Research Awareness and Application**

**Statement**

8. I understand the steps in conducting a scientific study.

9. I am aware of the importance of ethical approval and informed consent.

10. I know how to write a research hypothesis and define variables.

11. I am familiar with terms like PICO, PRISMA, and CONSORT.

12.I can critically evaluate a journal article.

13.I understand the difference between observational and interventional studies.

14. I have adequate knowledge of basic statistical terms (p-value, confidence interval, etc.)

**Section D: Attitudes & Feedback (Post-Only Section)**
*(Administer only after intervention)*

**Statement**

15. The curriculum improved my understanding of evidence-based dentistry.

16. I feel more confident in applying clinical decision-making after the program.

17. The hands-on research experience helped me learn better.

18. I would recommend this research training to others.

19. This module should be integrated into the regular BDS curriculum.

**Section E: Open Feedback (Optional)**
20. What was the most valuable part of this curriculum?
21. What challenges did you face during the program?
22. Any suggestions for improvement:
